# Socioeconomic Risk Factors for Pediatric Out-of-hospital Cardiac Arrest: A Statewide Analysis

**DOI:** 10.5811/westjem.59107

**Published:** 2023-04-28

**Authors:** Calvin Lukas Kienbacher, Guixing Wei, Jason Rhodes, Harald Herkner, Kenneth A. Williams

**Affiliations:** * The Warren Alpert Medical School of Brown University, Division of Emergency Medical Services, Department of Emergency Medicine, Providence, Rhode Island; † Medical University of Vienna, Department of Emergency Medicine, Vienna, Austria; ‡ Brown University, Population Studies and Training Center, Spatial Structures in the Social Sciences (S4), Providence, Rhode Island; § Rhode Island Department of Health, Center for Emergency Medical Services, Providence, Rhode Island

## Abstract

**Introduction:**

Economic hardship is a major threat to children’s health, implying that pediatric out-of-hospital cardiac arrest (pOHCA) might be promoted by lower incomes and child poverty. To target resources, it is helpful to identify geographical hotspots. Rhode Island is the smallest state by area in the United States of America. It has one million inhabitants and is comparable to many larger cities worldwide. We aimed to investigate the possible associations of pOHCA with economic factors and the coronavirus 2019 (COVID-19) pandemic. Our goal was to identify high-risk areas and evaluate whether the COVID-19 pandemic had an influence on delays in prehospital care.

**Methods:**

We analyzed all pOHCA cases (patients <18 years of age) in Rhode Island between March 1, 2018–February 28, 2022. We performed Poisson regression with pOHCA as dependent and economic risk factors (median household income [MHI] and child poverty rate from the US Census Bureau) as well as the COVID-19 pandemic as independent variables. Hotspots were identified using local indicators of spatial association (LISA) statistics. We used linear regression to assess the association of emergency nedical services-related times with economic risk factors and COVID-19.

**Results:**

A total of 51 cases met our inclusion criteria. Lower MHIs (incidence-rate ratio [IRR]) 0.99 per $1,000 MHI; P=0.01) and higher child poverty rates (IRR 1.02 per percent; P=0.02) were significantly associated with higher numbers of ambulance calls due to pOHCA. The pandemic did not have a significant influence (IRR 1.1; P=0.7). LISA identified 12 census tracts as hotspots (P<0.01). The pandemic was not associated with delays in prehospital care.

**Conclusion:**

Lower median household income and higher child poverty rate are associated with higher numbers of pediatric out-of-hospital cardiac arrest.

## INTRODUCTION

Children are among the most vulnerable populations in healthcare. Economic hardship has a significant burden on their well-being. The United Nations International Children’s Emergency Fund estimates that about a billion children live in poverty around the world.^1^ Their risk of dying during childhood is twice as high compared to individuals raised under economically stable conditions.^1^ In the United States of America (US), up to 20% of children are considered to be poor, which makes them one of the largest groups of destitute people in the country.^2^ The ongoing coronavirus 2019 (COVID-19) pandemic has further aggravated these issues globally.^3^

The American Academy of Pediatrics (AAP) defines children as individuals <18 years of age.^4^ Pediatric out-of-hospital cardiac arrest (pOHCA) is an infrequently encountered condition by emergency medical services (EMS).^5^, ^6^ An efficient rescue chain including prompt recognition of the condition, Basic Life Support instructions for bystanders prior to the arrival of EMS, proper training, and equipment for the EMS professionals are the cornerstones to improve outcomes, which nevertheless remain poor.^5^, ^7^, ^8^ To target resources, it might be useful for EMS personnel to know areas with higher likelihoods of encountering pOHCA. Identifying structural risk factors within society, such as economic hardship, might be of value for healthcare policymakers. Interventions, including first-aid training, community automatic defibrillator deployment, and prevention efforts may be of higher value in communities with higher prevalence.^9^

Rhode Island has approximately one million inhabitants and is the smallest state in the US by area. Its demographics and household economic status are near the national average.^10^ The US Census Bureau publishes the respective data on a census-tract basis (ie, small geographical areas with similar numbers of inhabitants).^11^ Rhode Island’s overall population density, with suburban and rural areas surrounding the densely populated urban area of the capital, Providence, makes it well comparable to many major cities worldwide. The state’s EMS system includes many different agencies but is regulated by the state Department of Health, which implies mandatory statewide EMS protocols and data reporting. All ambulance agency patient records are automatically uploaded into two databases, a biospatial platform and an EMS data capture tool (ImageTrend Elite, Lakeville, MN), where they are stored under the standardized paradigms of the National EMS Information System.^12^, ^13^ This comprises clinical and geospatial information on 911 emergency calls.

Geospatial data can be analyzed using various approaches. These include non-spatial methodology, such as linear and Poisson regression models, and methods of geospatial statistics. One of the latter techniques is the local indicators of spatial association (LISA) test, which focuses on reviewing geographical areas (eg, census tracts) in the context of their surroundings regarding a characteristic (eg, rate of EMS calls).^14^ It allows identification of clusters with similar properties or outliers with dissimilar properties in immediate neighborhoods. The global Moran’s I is another geospatial test, which facilitates the detection of geographical patterns over the entire map of interest.^15^ Similar methods have been used in the past to examine the geospatial properties of adult OHCA.^16^–^18^

**Population Health Research Capsule**

What do we already know about this issue?

*Pediatric out-of-hospital cardiac arrest (pOHCA) is a rare but important condition in emergency medicine. Children’s health is associated with socioeconomic status*.

What was the research question?

*Is there an association between median household income and the occurrence of pOHCA?*

What was the major finding of the study?

*Lower median household income and higher child poverty rates are associated with higher numbers of pOHCA (1% per 1,000$ change; P=0.01)*.

How does this improve population health?

*Interventions focusing on areas with lower household incomes might help to improve patient care and the prevention of pOHCA*.

We aimed to investigate whether there is an association between a census tract’s median household income (MHI), child poverty rate, and the occurrence of pOHCA over a four-year observation period that included two years prior to the COVID-19 outbreak and the first two years of the pandemic. Furthermore, we aimed to elaborate on whether the pandemic was associated with any EMS-related delays of patient care. To our knowledge, no study has yet investigated these issues using a comprehensive, statewide dataset.

## METHODS

We conducted a retrospective analysis of all ambulance calls for pOHCA in the state of Rhode Island between March 1, 2018–February 28, 2022. Subjects had to be <18 years of age, according to the AAP’s definition of childhood.^4^ The study period corresponds to equal intervals before (March 1, 2018–February 29, 2020) and after (March 1, 2020–February 28, 2022) the beginning of the COVID-19 pandemic.

Records of non-primary responses (eg, interfacility transfers) and mass casualty incidents were excluded. We extracted the patients’ demographics (gender and age) and EMS-related information (geo-coordinates, mission times, clinical data, and the patient report narrative) from the biospatial and EMS data capture platforms. The datasets from these two sources were merged in Microsoft Excel 16.62 (Microsoft Corporation, Redmond, WA) based on the patient-care report number, a unique identifier. All patient care reports including their narratives were manually reviewed by a board-certified EMS physician, who is also a licensed paramedic.

The census tracts’ most recent year (2020) shapefiles, demographic (population <18 years and poverty rates), and economic (median household income [MHI]) information were downloaded from the website of the US Census Bureau.^19^, ^20^ We excluded census tracts without any inhabitants <18 years of age (ie, no population at risk, such as water areas, of which there are four). Another two census tracts, which are inhabited but isolated islands, were excluded from geospatial analysis only, as they do not have any adjacent neighbors according to our definition (identifiers of census tracts comprising water: 44005990000, 44009990100, 44009990200; islands: 44005041300, 44009041500). The Providence Airport census tract (identifier 44003980000) was merged with one of its neighbors (identifier 44003021902) for geospatial analysis only, as it doesn’t have any sociodemographic attributes.

The data was imported into ArcGIS Pro 2.9.3 (Esri Corporation, Redlands, CA). All pOHCA cases were assigned to census tracts by their geocoordinates. We calculated rates (ie, the number of pOHCA cases over the four-year observation period divided by the population <18 years of age) for all census tracts and the state of Rhode Island. We investigated differences in MHI, measured in 2020 inflation-adjusted US dollars ($), using a Poisson regression model with the number of pOHCA cases as the dependent and the economic risk factors MHI and child poverty rate, as well as the period of the pandemic, as independent variables. We used the *poisson* command in Stata 17MP (StataCorp, LLC, College Station, TX), with the *exposure* option to control for the census tracts’ individual population size. We used the *vce* [cluster clustvar] option to link census tracts by their geographic identifiers, thereby allowing us to include COVID-19 (before pandemic and pandemic phase) as a variable into the model. Results are presented as incidence-rate ratios (IRR) with their corresponding 95% confidence intervals and *P*-values. We controlled for the population at risk.

We calculated Global Moran’s I statistics across all eligible census tracts. LISA statistics regarding the pOHCA rate of each census tract were calculated using a row-standardized, with 499 permutations.^21^ We used the spatial contiguity Queen criterion (sharing common edges and/or corners) to define the neighbor relationship. Results are presented graphically on a map.

We defined EMS times as follows:

- Response time: from unit dispatched to arrival at the patient
- On-scene time: from arrival at the patient to leaving the scene
- Transport time: from leaving the scene to arrival at the destination
- Back-to-service time: from patient arriving at destination to unit back to service
- Overall mission time: from dispatch to unit back in service.

Time values of zero were excluded. We calculated differences in medians between before and after the beginning of the COVID-19 pandemic and their corresponding 95% CIs, using quantile regression in Stata 17MP. We used linear regression models with the respective times as the dependent variables. Census tracts neighborhood hotspots (ie, high-low outliers identified by LISA) and the economic risk factors served as independent variables. A two-sided *P*-value ≤0.05 was considered to be statistically significant.

As data on the geospatial distribution of EMS calls for pOHCA is sparse, we could not perform a formal a priori sample-size calculation. We therefore chose to include all cases within the observation period into our study. The Ethics Committee of the Rhode Island Department of Health approved the study protocol with an exemption from full review (vote #2022-01). Our study was conducted following the principles of the Declaration of Helsinki.

## RESULTS

A total of 51 emergency calls, of which 24 were for females (47%) met our inclusion criteria. Twenty-four cases (47%) occurred before and 27 (53%) after the beginning of the COVID-19 pandemic. The median age of the study population was two years (interquartile range [IQR] 0–13). Table 1 provides the baseline characteristics of the study population.

The associated median MHI was $62,589 (IQR 39,754–$75,591) and the median child poverty rate 22.1% (IQR 4.9–32.9%). The statewide averages were $71,166 (IQR $51,349–90,795) and 8.8% (IQR 1.4–23.1%), respectively. We identified four census tracts without any population <18 years of age. In 45 census tracts, pOHCA rates ranged from 6–30 per 10,000 children. All other census tracts (201) did not observe any cases of pOHCA. We found an overall rate of pOHCA of 1 per 4,000 individuals <18 years of age over the four-year observation period in Rhode Island.

**Figure 1:**
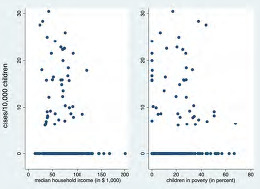
Association between the rate of pediatric out-of-hospital cardiac arrest, median household income of the prior 12 months in 2020 United States dollars, and child poverty.

Our Poisson regression analysis showed that lower MHI was associated with higher numbers of pOHCA (IRR 0.99, 95% CI 0.976–0.997), for every $1,000 change in MHI; *P*=0.01). The same applied to higher child poverty rates (IRR 1.02, 95% CI 1.004–1.03], for every percentage change in child poverty rate; *P*=0.02) (see Figure 1). The pandemic had no significant association with the occurrence of pOHCA (IRR 1.1, 95% CI 0.6–2; *P*=0.7).

**Figure 2:**
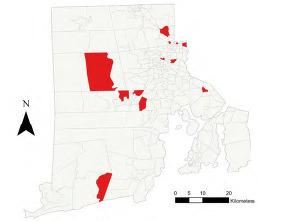
High-low outlier census tracts (red) in respect of the rate of pediatric out-of-hospital cardiac arrest in Rhode Island, arrow indicates north. *N*, north.

No cases were lost by excluding six census tracts due to their geographical properties. In the 244 census tracts included for geospatial analysis, Global Moran’s I statistics showed a random spatial pattern of pOHCA rates across Rhode Island over the four-year observation period (z-score= −0.08, *P*=0.93). Focusing on immediate neighborhoods, LISA analysis revealed that 12 census tracts were significant high-low outliers (*P*<0.01), ie, community hotspots with high rates, surrounded by census tracts with relatively low rates. No low-high outliers, high-high, or low-low clusters were identified (Figure 2). The high-low clusters were concentrated in the northern parts of Rhode Island. This includes the less wealthy regions of the greater city area of Providence, in the northeast, and suburban Kent County in the northwest. The wealthier Newport and Washington counties in the southeast and southwest had cases of pOHCA but hardly any clusters in immediate neighborhoods.

Regarding EMS times, one case (search for a missing child) was excluded from the analysis. In the remaining 50 missions, we did not find any significant differences between the time periods before and since the beginning of the COVID-19 pandemic in response (median difference 0, 95% CI −1–2 minutes), on-scene (median difference −2, 95% CI −13–9 minutes), transport (median difference −2, 95% CI −6–2 minutes), back-to-service (median difference −4, 95% CI −29–21 minutes), or overall mission time (median difference −9, 95% CI −40–21 minutes). Table 2 summarizes our secondary findings. The transport time was log transformed to better fit the linear regression model. Higher MHI was associated with longer transport times (coefficient 0.01, 95% CI 0.003–0.019), for every $1,000 change) and higher child poverty rates (coefficient −0.01, 95% CI −0.024–0.002], for every percentage change) were associated with shorter transport times. The EMS times were not altered by census tracts being community hotspots.

## DISCUSSION

Our findings indicate that pOHCA might more frequently occur in census tracts with lower MHI and higher child poverty rates. While the overall distribution of cases follows a random geographical pattern, we were able to identify hotspots on the community level using geospatial analysis techniques. These also concentrate around the less wealthy neighborhoods.

Strengths of our study include the analysis of comprehensive, statewide data over a four-year observation period by a multidisciplinary team. The analyses were conducted by experts in the field and controlled for the population at risk. Furthermore, we are confident that Rhode Island is a good model region for our analyses due to its sociodemographic profile. We did not limit our research question to the whole state; rather, we also took into account differences of immediate neighborhoods. These include urban and suburban areas, which many cities worldwide have.

Most of the previouly published literature on the investigation of socioeconomic risk factors and OHCA focuses on adults. Prior research strongly supports the concept that a higher socioeconomic status is protective in this context, with the outcome being survival.^22^, ^23^ Data on the situation in children is still sparse. One trial examined the influence of parental socioeconomic status on the survival of pOHCA, indicating that higher household income and education increase the chances of survival.^24^ Our findings are also well compatible with those of Salmi et al, who found that children living in poorer economic areas are in general more likely to be encountered by EMS than those living in wealthier areas.^25^ However, their study includes many disease entities, not solely cardiac arrest.

A few studies aimed to identify geographical hotspots of OHCA using similar means of geospatial statistics. Those studies focused on adults on a census-tract level and show feasibility of the methodology in this context.^16^–^18^ Wong et al included cases of pOHCA but provided no subgroup analysis for the pediatric population.^26^ Our results are consistent with those of Sasson and colleagues, who also found that areas with higher numbers of OHCA tend to have lower household incomes.^17^ Targeted interventions, including the education of potential bystanders, public access defibrillators and naloxone boxes might improve the care of patients in high-risk areas. Enhancing the economic situation in less wealthy regions, eg, by subsidies and support for unemployed parents, might help to prevent the condition in the first place. Furthermore, broad insurance coverage for regular health care checks for all children would be desirable to detect chronic medical conditions, which increase the risk of early pOHCA.

Interestingly, we did not find any differences in EMS times between the periods before and since the beginning of the COVID-19 pandemic. Contrarily, prior data indicates that on-scene times might be longer since its onset.^27^ However, those authors looked at all ambulance missions with lights and sirens, not solely at cardiac arrest, in which the treatment algorithms are well standardized.^27^ Other factors might be system-specific, such as how long it takes to find the right destination for the individual patient before initiating transport.

Prior research also shows that respiratory issues are the most common reason for cardiac arrest in children, with primary heart problems being rare.^7^ Noteworthy, shockable rhythms were more common in our population (12%) than expected. The reasons for this discrepancy between the literature and our data remain unclear. Screening of the patient-care report narratives revealed no clearly documented reasons for the cardiac arrest in most of our cases.

## LIMITATIONS

Our study has limitations. We used the medians of the 2020 median household incomes of the census tracts as an economic risk factor. This seemed the most appropriate strategy to us, as individual earnings remain unknown. One must, therefore, bear in mind that a census tract’s MHI might differ from that of the family of an individual case. The same applies to the child poverty rate. However, our approach to use census tract-level data is consistent with previously published research and has also been used in the field of cardiovascular medicine.^28^–^31^ Furthermore, we took the most recently available (2020) five-year census data as the basis for our calculations. This information might not necessarily reflect the economic situation before or after this year. We believe that incorporating both MHI and the child poverty rate into our model increases the interpretability of the findings.

## CONCLUSION

Our results indicate that EMS crews serving the population of less wealthy census tracts might be more likely to face pediatric out-of-hospital cardiac arrest. This finding could have implications for targeted professional and bystanders’ training, as well as community interventions. Prevention strategies might include regular healthcare checks in children and approaches to enhance the economic situation of a region, eg, with subsidy programs. Data on the topic is still scarce.

## Figures and Tables

**Table 1:** Baseline characteristics of the study population.

	Pediatric out-of-hospital cardiac arrest cases (N=51)
Female, n (%)	24 (47)
Age, years, median (IQR)	2 (0 to 13)
Before COVID-19, n (%)	24 (47)
Shockable rhythm, n (%)	6 (12)
Biological death on arrival of EMS, n (%)	4 (8)

*IQR*, interquartile range; *COVID-19*, coronavirus 2019; *EMS*, emergency medical services.

**Table 2:** Emergency medical service-related times before and since the beginning of the COVID-19 pandemic.

	Overall	Before pandemic (n=24)	Since pandemic (n=27)	Before vs since pandemic, median [95% CI]
Response time, minutes, median (IQR), n=49	5 (4 to 6)	5 (4 to 7)	4 (3 to 5)	0 [−1, 2]
On-scene time, minutes, median (IQR), n=47	14 (7 to 28)	13 (7 to 24)	16 (8 to 30)	−2 [−13, 9]
Transport time, minutes, median (IQR), n=46	8 (5 to 11)	7 (5 to 10)	9 (6 to 11)	−2 [−6, 2]
Back-to-service time, minutes, median (IQR), n=46	63 (46 to 83)	60 (40 to 78)	64 (53 to 85)	−4 [−29, 21]
Overall mission time, minutes, median (IQR), n=50	91 (67 to 121)	83 (55 to 105)	92 (76 to 127)	−9 [−40, 21]

*IQR*, interquartile range.
